# Bleeding Classifications in CABG: perspective on Prognostic Performance

**DOI:** 10.21470/1678-9741-2020-0034

**Published:** 2020

**Authors:** Rohan Magoon, Souvik Dey, Jasvinder Kaur Kohli, Ramesh Kashav

**Affiliations:** 1 Department of Cardiac Anaesthesia, Atal Bihari Vajpayee Institute of Medical Sciences (ABVIMS) and Dr. Ram Manohar Lohia Hospital, Baba Kharak Singh Marg, New Delhi, India. E-mail: rohanmagoon21@gmail.com

**To The Editor,**

Perioperative bleeding following cardiac surgery predisposes to poor outcomes by incurring an elevated susceptibility to infection and organ injury (renal insult, myocardial injury, transfusion-associated lung injury, and stroke), intricately affecting the length of intensive care unit (ICU) and hospital stay and the associated morbidity-mortality^[1]^. Albeit a considerable clinical impact, sound risk-stratification of the complication is largely precluded by the heterogeneity of the bleeding classifications and the subsequent validity in predicting patient outcomes.

A variety of bleeding classifications have been proposed and evaluated in cardiovascular clinical trials with the majority of the literature emanating from the setting of acute coronary syndromes (ACS). There is a recent clinical interest to evaluate the prognostic significance of different bleeding definitions in patients undergoing coronary artery bypass grafting (CABG) considering the sizeable frequency of ACS patients (nearly 12%) undergoing surgical revascularization during index hospitalization, predisposed patient cohort (comorbid status and preoperative anticoagulant therapy), and the need to tenuously balance the risk of perioperative bleeding and thrombosis^[2]^. The recent inclusion of CABG-related bleeding (Type-4) in the Bleeding Academic Research Consortium (BARC) 2011 consensus document^[2]^ bears testimony to the aforementioned fact (Table 1).

**Table 1:** The BARC definition of CABG-related bleeding (Type 4).

• Perioperative intracranial bleeding (within 48 hours)
• Reoperation post-sternotomy closure (for alleviating bleeding)
• Transfusion of 5 U whole blood or packed red blood cells (restricted to allogenic transfusion within 48 hours)
• 2 L chest tube output (within 24 hours)

BARC=Bleeding Academic Research Consortium; CABG=coronary artery bypass grafting

Dyke et al.^[3]^ proposed a Universal Definition of Perioperative Bleeding (UDPB) stratifying perioperative bleeding risk (0: insignificant; 1: mild; 2: moderate; 3: severe; 4: massive) based on nine parameters (delayed sternal closure, chest-tube output, packed red blood cell [PRBC], fresh frozen plasma [FFP], platelet [PLT], cryoprecipitate transfusion, use of prothrombin complex concentrates [PCC], recombinant activated factor VII, and need of surgical re-exploration). Kinnunen et al.^[4]^ outlined an increased inotropic requirement and ICU stay, elevated risk of renal replacement therapy, stroke, low-cardiac output, and inhouse mortality in association with an advanced UDPB Class 3-4 in their retrospective evaluation of 2,764 post-CABG patients. The European registry of CABG (E-CABG) investigators also developed a bleeding severity definition (Grade 0: lack of use of blood products with exception of 1 U PRBC; Grade 1: transfusion of PLT, FFP, PCC, or 2-4 U PRBC; Grade 2: transfusion of 5-10 U PRBC or reoperation for bleeding; Grade 3: > 10 U PRBC transfusion)^[5]^.

Brascia et al.^[6]^ compared the prognostic performance of UDPB, E-CABG, the Study of Platelet Inhibition and Patient Outcomes (PLATO), Clopidogrel and Aspirin Optimal Dose Usage to Reduce Recurrent Events-Seventh Organization to Assess Strategies in Ischemic Syndromes (CURRENT-OASIS7), SafeTy and Efficacy of Enoxaparin in Percutaneous coronary intervention patients, an internationaL randomized Evaluation (STEEPLE), Efficacy and Safety of Subcutaneous Enoxaparin in Non-Q Wave Coronary Events (ESSENCE) for predicting stroke, early mortality, acute kidney injury stage 3, and sternal wound infection in the prospective multicentric evaluation of 3,730 patients undergoing CABG. They described that the predictive ability of UDPB, E-CABG, PLATO, and CURRENT-OASIS7 was higher compared to the STEEPLE and ESSENCE bleeding classifications^[6]^. Interestingly, a retrospective comparison of prognostic performance of E-CABG, BARC, UDPB, and PLATO following off-pump CABG by Xi et al.^[1]^ revealed a superior categorization of outcomes with E-CABG, BARC, and UDPB compared to PLATO classification.

A number of caveats surface on a meticulous evaluation of the recent literature on the prognostic value of the bleeding classifications in CABG-setting. First and foremost, it is clinically challenging to characterize prognostically relevant perioperative bleeding, particularly in on-pump CABG. Moreover, the distinguished transfusion thresholds and rates of reoperation for bleeding compound the situation furthermore. Secondly, strictly speaking, BARC Type 4 bleeding is the lone specific CABG-related bleeding classification. It is noteworthy that a decline in the hematocrit is an integral component of bleeding classification in non-surgical setting (PLATO, etc.), whereas the inclusion of the interventions aimed at alleviating ongoing bleeding in the surgical bleeding definitions (UDPB, BARC, E-CABG, etc.) probably account for an augmented prognostic perioperative performance. Lastly, majority of the available literature in this area is retrospective in nature.

To conclude, there is a recent emphasis on the evaluation of prognostic value of various bleeding classifications for an improved characterization of CABG-related bleeding. An augmented standardization in conjunction with validation across diverse data sets can further refine the definition of the risk of perioperative bleeding and the modulation of the strategies aimed at minimizing the impact on the postoperative outcomes.
